# Stability at the half pin–frame interface on external fixation constructs

**DOI:** 10.1007/s11751-016-0269-z

**Published:** 2016-10-13

**Authors:** Alexios Dimitrios Iliadis, Parag Kumar Jaiswal, Jay Meswania, Gordon Blunn, David Goodier, Peter Calder

**Affiliations:** 1 Limb Reconstruction Unit, Royal National Orthopaedic Hospital NHS, Brockley Hill, Stanmore, Middlesex HA7 4LP UK; 2 University College London - Institute of Orthopaedics and Musculoskeletal Science, Royal National Orthopaedic Hospital (RNOH), Brockley Hill, Stanmore, HA7 4LP UK

**Keywords:** Half pin–frame interface, External fixation constructs, Grub screws, Bolt screws, Stability

## Abstract

A mechanical study investigating the use of two different methods (grub and bolt screws) to secure external fixation half pins to circular frames. A four part experiment: (1) Grub and bolt screws were used to secure half pins in Taylor Spatial frames. Loosening torques were measured using a calibrated torque wrench. (2) Using universal testing machine (UTM), axial loading was applied to establish thresholds for loosening in grub and bolt screw constructs. (3) We established the application torque to produce failure at the head–driver interface using these two methods. (4) Grub and bolt screw constructs were created controlling torque. Using UTM, axial loading was applied to establish thresholds for loosening. Statistical analysis was conducted using SPSS v20.0.0. (1) Higher torque is employed when bolt rather than grub screws is used to secure half pins on Rancho cubes (*p* < 0.05). (2) Loading threshold for loosening is higher in bolt screw constructs when the torque applied to secure the constructs is not controlled (*p* < 0.05). (3) Torque required for failure at the head–driver interface was 5.3 Nm for grub screws and 9.9 Nm for bolts. (4) Loading threshold for loosening is higher in grub screw constructs when the same torque was applied to secure them (*p* < 0.05). Bolt screws can be employed to secure the half pin–frame interface. They offer good stability and reduce failure at the head–driver interface. Further research is needed to determine the mechanical properties of such constructs in vivo.

## Introduction

The Ilizarov method has proved successful in the treatment of a wide spectrum of orthopaedic disorders [1]. The success of this method is to be attributed to the combination of the biomechanics of the external fixation apparatus and the biological principles of distraction osteogenesis [1]. The stability of the external fixation apparatus is critical in preventing excessive movement which could increase morbidity and compromise bone healing [2, 3].

Half pins were introduced to address some of the disadvantages of the conventional apparatus which consisted of fine wires only. There are contested benefits in reducing soft tissue transfixation so allowing for less morbidity and increased mobility [4]. Furthermore, there is simplicity with regard to insertion in anatomically challenging areas, a reduction in fixation time and lower risk of complications [5].

High stresses at the pin–bone interface contribute to micro-motion and failure resulting in unicortical loosening. Experimental models have demonstrated far higher pressures are generated under loading conditions at the bone interface from half pins as compared to fine wires [5]. The pin–bone interface has therefore been regarded as the weakest link in the mechanical stability of external fixation systems and has been investigated extensively [6–8]. In contrast, no studies have looked at the interface between the half pin and frame assembly. Loss of stability here compromises the bone remodelling process through a change in the biomechanics of the construct. In the systems of Ilizarov and Taylor Spatial frames (TSF) external fixation by Smith and Nephew, grub screws are used to secure half pins on Rancho cubes. The aim of this study is to investigate 2 different methods used (grub screw or 10 mm stainless steel hexagonal headed bolt [M6 A2-70]) to secure external fixation half pins to circular frames. This is to determine whether use of bolts is appropriate and could reduce the potential for loosening at this interface.

## Materials and methods

All participating clinicians were members of the Limb Reconstruction Unit at the Royal National Orthopaedic Hospital with experience in the assembly and application of external fixation systems. Two of the authors participated. The remaining participants were blinded as to the purposes of the study.

**Fig. 1:**
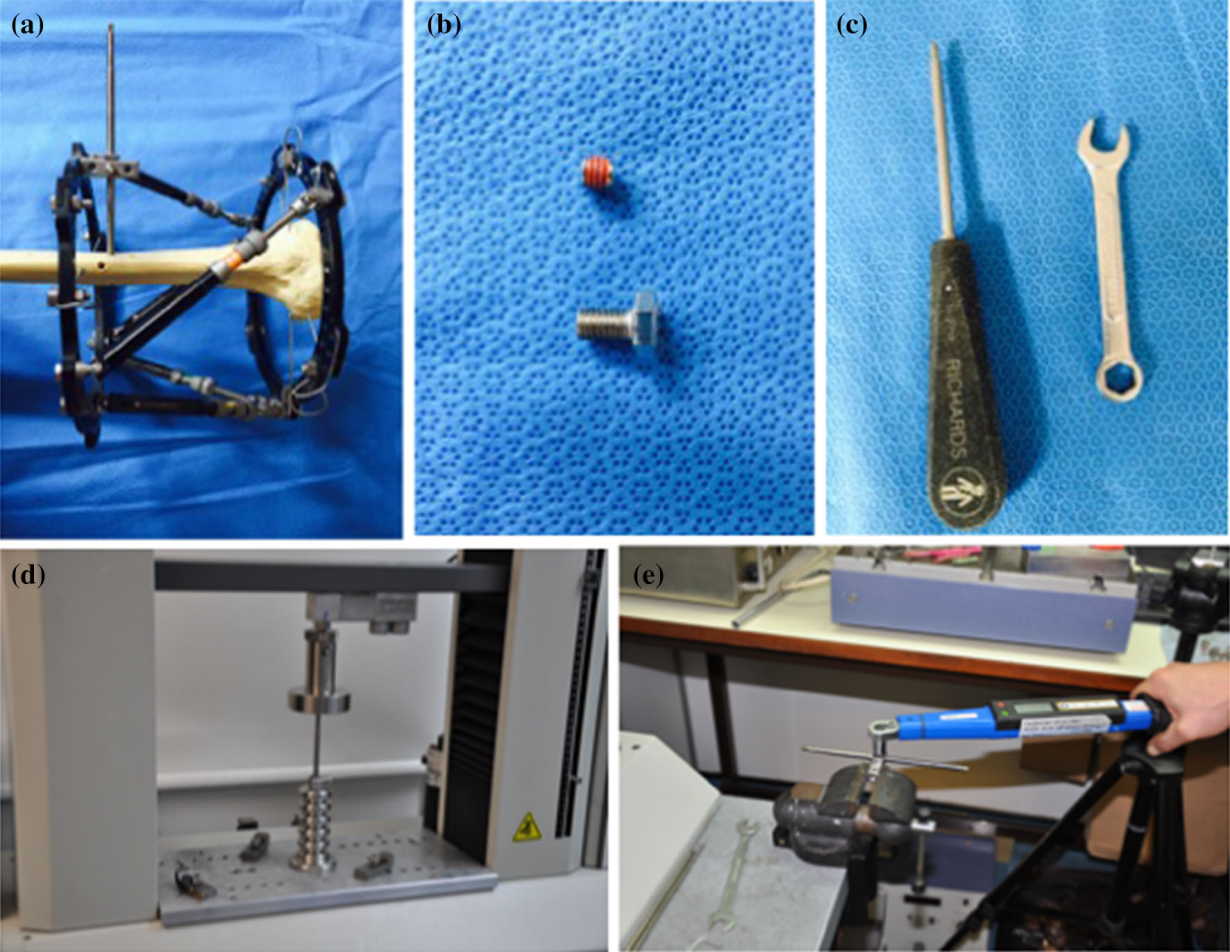
Form* top left clockwise*: **a** TSF mounted on saw bone, **b** set and bolt screws, **c** wrench and straight hex driver, **d** universal testing machine and **e** calibrated torque wrench

In an attempt for mounting conditions to resemble those in the operating room, hybrid external fixation frames (Taylor Spatial frames (TSF)—Smith and Nephew, Memphis, TN) were constructed and mounted on saw bones (Fig. 1a), and appropriate instruments available from the TSF set were utilized exclusively. They consisted of two 180-mm rings connected by fast struts and secured with two tensioned wires and one half pin per ring. A universal testing machine at the University College London Institute of Orthopaedics and Musculoskeletal Science was used, under the supervision of an experienced technician, to apply axial loading on the constructs.

The experiment was conducted in four parts:

### Part 1

We sought to determine whether there was a difference in the torque applied for securing half pins to Rancho cubes when employing these two methods; the mounting conditions were similar to those encountered in clinical practice. Five participants were asked to secure half pins on Rancho cubes using 5 grub (set) screws and 5 standard 10-mm bolts (Fig. 1b) in an alternating fashion to account for fatigue. They were instructed to apply as much torque as they would in clinical practice to the point they were satisfied that the half pin has been secured adequately in the Rancho cube. New Rancho cubes and screws were used on each occasion to avoid threads cutting out and altering the results. Wrenches were used to tighten the standard 10-mm bolts and straight hex drivers for the grub screws as would be the case when done intra-operatively (Fig. 1c).

Using a calibrated torque wrench (Torqueleader TWD20 Torque Wrench—MSK/EQ/40 Calibration 03/02/2012), the torque (Nm) required to loosen; the screws was measured for each construct.

### Part 2

We sought to examine which of the two methods held the half pin better; this was tested in loading similar to those encountered in clinical practice. Two participants were each asked to secure 5 half pins using grub screws and 5 half pins using standard 10-mm bolts in the same manner as the first part of our experiment. The Rancho cubes were released from the rings, and the whole half pin–Rancho cube construct was removed from the saw bone using T-handles. The constructs were then placed on a universal testing machine—UTM (Fig. 1d), and increasing axial loading was applied to determine loosening points. This was determined as mechanical failure on the load deformation curve and was associated with loosening at the interface between the pin and Rancho cube.

### Part 3

We sought to determine the tightening torque that can be applied safely (prior to breakage) at the head–driver interface when using these two methods. Twenty grub screws and twenty bolts were used to secure half pins on Rancho cubes using a calibrated torque wrench to determine the point at which breakage at the driver–head interface occurs (Fig. 1e).

### Part 4

We sought to examine, when a controlled torque was applied for securing all half pins, the loosening points of these constructs when subjected to axial loading. From the investigation in part 3, it was established that 5 Nm was a safe amount of torque to be applied on grub screws prior to breakage at the driver–head interface. Ten constructs using grub screws and ten using bolts were secured applying 5 Nm with a calibrated torque wrench. The constructs were then placed on a universal testing machine—UTM (Fig. 1d), and increasing axial loads were applied to determine points of loosening as determined as mechanical failure on the load deformation curve.

The same process was followed for ten bolt constructs secured using 9.5 Nm torque which, in part 3, was found to be a safe amount of torque prior to breakage at the driver–head interface.

### Statistics

SPSS 20 was used to perform statistical analysis. Our data distribution was assessed for normality using the Shapiro–Wilk test. The *t* test was used for parametric data and Mann–Whitney *U* test for nonparametric data. Statistical significance was determined as *p* values of <0.05.

## Results

### Part 1

**Fig. 2:**
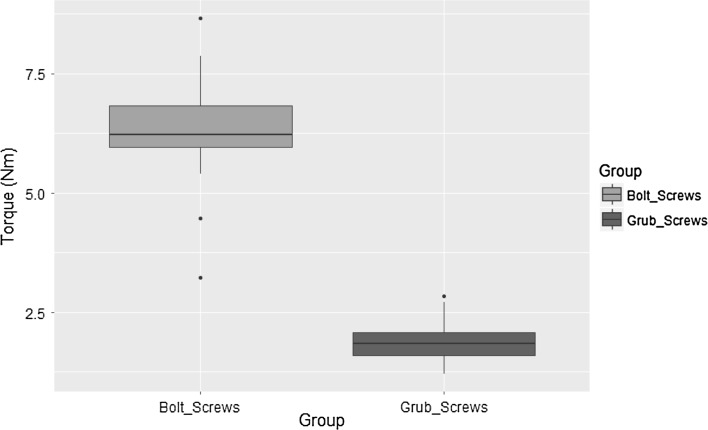
*Box plot* demonstrating loosening torque values (Nm) for bolt and grub screw constructs

Figure 2 demonstrates the loosening torque values obtained in the first part of our experiment. The values obtained for bolts were higher (median 6.3 SD 1.1) than grub screws (median 1.84 SD 0.4). The Shapiro–Wilk test confirmed both our data sets are normally distributed. A *t* test confirmed the statistical significance (*p* < 0.05).

### Part 2

Table 1 demonstrates the loads required to produce loosening at the half pin–Rancho cube interface when ten constructs with grub screws and ten constructs with bolts were subjected to increasing axial loading on UTS. The Mann–Whitney test was employed for statistical analysis as determined appropriate by Shapiro–Wilk test of our data distribution. Significantly higher loads were required for loosening to occur on the construct using bolts (*p* < 0.05).

**Table 1:** Loads required on UTS for loosening at the half pin–Rancho cube interface for grab screw and bolt screw constructs mounted without controlling torque

Group	*N*	Min (N)	Q25 (N)	Median (N)	Mean (N)	Q75 (N)	Max (N)	SD (N)
BS	10	1799.9	2010	2102.8	2032.7	2103.6	2104.4	122.5
GS	10	949	1084.6	1368.4	1281.6	1393.8	1674.6	226.3

### Part 3

**Fig. 3:**
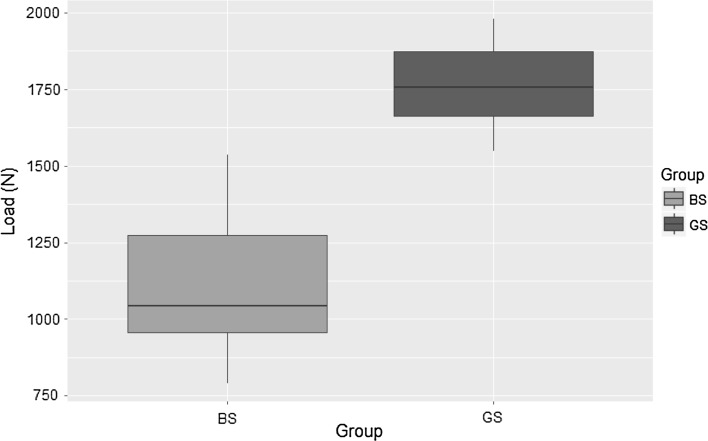
Axial loads (N) required on UTM for loosening at the half pin–Rancho cube interface for bolt screw (BS) and grub screw (GS) constructs secured with a torque of 5 Nm

The mean torque applied for breakage to occur at the driver–head interface when using grub screws was 5.31 Nm (SD 0.19). In every case, breakage occurred at torque values >5 Nm when using bolts this was 9.92 Nm (SD 0.15). In every case, breakage occurred at torque values >9.5 Nm (Fig. 3).

### Part 4

**Fig. 4:**
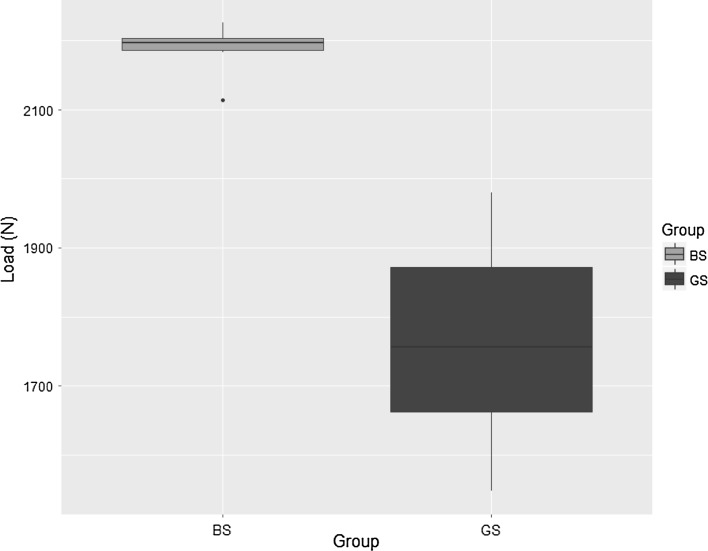
Axial loads (N) required on UTM for loosening at the half pin–Rancho cube interface for bolt screw (BS) and grub screw (GS) constructs secured with a torque of 9.5 and 5 Nm, respectively

When comparing axial loads to loosening in grub screw constructs secured with a 5-Nm torque and those on the bolt constructs secured without torque control, there was a statistically significant difference in favour of bolt constructs (Mann–Whitney *U* test, *p* < 0.05). This is shown in Table 2. When comparing the values obtained for grub screws tightened with a torque of 5 Nm with those obtained for bolts tightened at torque of 9.5 Nm, significantly higher axial loads can be applied before loosening on the bolt constructs (Mann–Whitney test). This is shown in Fig. 4.

**Table 2:** Axial loads required for loosening at the half pin–Rancho cube interface for grab screw constructs using 5-Nm torque to secure and bolt screw constructs mounted without controlling torque

Group	*N*	Min (N)	Q25 (N)	Median (N)	Mean (N)	Q75 (N)	Max (N)	SD (N)
BS	10	1799.9	2010	2102.8	2032.7	2103.6	2104.4	122.5
GS	10	1547.9	1662.9	1757	1766.4	1871.9	1979.9	148.9

## Discussion

We have simulated clinical conditions by use of saw bone models; we recruited subjects with experience in mounting frames and instructed them to assemble these constructs in a manner similar to their clinical practice. In the first part of the experiment, loosening torque values were determined. These are not the same as the tightening torque values as different forces are involved (static vs dynamic). Values obtained demonstrate statistically significant higher loosening torque values when bolts are used to secure half pins. This suggests a higher torque is applied when half pins are secured with bolts. We surmise the difference is due to a wrench (spanner) being used in contrast to the straight hex driver for grub screws. The axial compression force (clamp force) applied through either bolt or grub screw would determine the security of hold at the half pin–Rancho cube interface. This clamp force is affected by many other variables too: the bolt diameter. The type and number of threads on the bolt, the bolt material and the torque applied. The last variable is that under control by the surgeon and may influence the likelihood of these constructs to failure.

In the second part of the experiment, we demonstrated that, under such mounting conditions, significantly higher axial loading forces are required for loosening to occur at the half pin–Rancho cube interface when bolts are used instead of grub screws. Failure in our experiments was defined as mechanical failure on the load deformation curve, and this was associated with loosening at the half pin–Rancho cube interface. Based on these results, we can demonstrate that bolts hold the half pins equally or better than grub screws in experimental conditions of loading to failure.

The results obtained in the first two parts of the experiment suggest that higher stability is offered by bolts; this may be from the use of wrenches for securing the half pins with higher torques and clamp forces applied.

Hex wrenches and Allen keys are available in the supplied instrument sets for mounting half pins in Rancho cubes. In our experience, they are associated with a risk of breakage at the head–driver interface, causing difficulties should the fixator assembly need to be dismantled. The third part of the experiment showed that higher torques can be applied safely when using bolts.

In the fourth part, we controlled the torque applied when securing these fixator constructs. The results suggested that, when equal torque is applied, grub screws are superior in providing stability in axial loading. The grub screw point profile may produce a better grip on the half pin than that on the bolt when the same torque is used, but when both types of screws are mounted applying maximum torque, bolts demonstrate a significantly higher threshold for loads prior to loosening. This reinforces the clinical practice to increase tightening torque in application so that an increase in clamp force is achieved and correspondingly the tolerance to load to failure.

These results demonstrate that bolts achieve good stability at the half pin–Rancho cube interface by tolerating higher axial loads than grub screws before loosening. This is a result from the greater torque that can be produced using a spanner or wrench. Whilst the point profile of the grub screw secures a better hold of the half pin when the torque used is equal, the driver used to insert the grub screw is limited in delivery of a high maximum torque before breakage; this appears to be the limiting factor for the security of hold on the half pin by grub screws.

We conclude that bolts can be employed safely to secure half pins in Rancho cubes and, if tightened maximally, provide as good or better security of hold on the pin to grub screws.
